# Regulation of intestinal hPepT1 (SLC15A1) activity by phosphodiesterase inhibitors is via inhibition of NHE3 (SLC9A3)

**DOI:** 10.1016/j.bbamem.2007.04.006

**Published:** 2007-07

**Authors:** Catriona M.H. Anderson, David T. Thwaites

**Affiliations:** Epithelial Research Group, Institute for Cell and Molecular Biosciences, Faculty of Medical Sciences, University of Newcastle upon Tyne, Newcastle upon Tyne, NE2 4HH, UK

**Keywords:** PepT1, Dipeptide transport, Na^+^/H^+^ exchange, NHE3, Intestinal absorption, Drug transport

## Abstract

The H^+^-coupled transporter hPepT1 (SLC15A1) mediates the transport of di/tripeptides and many orally-active drugs across the brush-border membrane of the small intestinal epithelium. Incubation of Caco-2 cell monolayers (15 min) with the dietary phosphodiesterase inhibitors caffeine and theophylline inhibited Gly–Sar uptake across the apical membrane. Pentoxifylline, a phosphodiesterase inhibitor given orally to treat intermittent claudication, also decreased Gly–Sar uptake through a reduction in capacity (*V*_max_) without any effect on affinity (*K*_m_). The reduction in dipeptide transport was dependent upon both extracellular Na^+^ and apical pH but was not observed in the presence of the selective Na^+^/H^+^ exchanger NHE3 (SLC9A3) inhibitor S1611. Measurement of intracellular pH confirmed that caffeine was not directly inhibiting hPepT1 but rather having an indirect effect through inhibition of NHE3 activity. NHE3 maintains the H^+^-electrochemical gradient which, in turn, acts as the driving force for H^+^-coupled solute transport. Uptake of β-alanine, a substrate for the H^+^-coupled amino acid transporter hPAT1 (SLC36A1), was also inhibited by caffeine. The regulation of NHE3 by non-nutrient components of diet or orally-delivered drugs may alter the function of any solute carrier dependent upon the H^+^-electrochemical gradient and may, therefore, be a site for both nutrient–drug and drug–drug interactions in the small intestine.

## 1 Introduction

The human intestinal H^+^-coupled di/tripeptide transporter (hPepT1 or SLC15A1) is responsible for the transport of a significant proportion of dietary protein across the brush-border membrane of the small intestinal epithelium. hPepT1 is also of pharmacological interest as it can transport orally-active drugs such as β-lactam antibiotics and angiotensin converting enzyme inhibitors [1–4]. More recently a number of amino acid modified pro-drugs such as val-acyclovir, l-DOPA-l-Phe and the anti-hypotensive midodrine have been designed specifically to target hPepT1 and thus to enhance oral bioavailability [3–5].

When expressed heterologously, hPepT1 functions as a Na^+^-independent, pH-dependent, H^+^-coupled transporter [6,7]. However, until the mid-1980s, it was considered widely that di/tripeptide transport was via a Na^+^-coupled transport mechanism. Indeed, dipeptides can stimulate Na^+^ absorption in the human small intestine and Na^+^ uptake in human intestinal Caco-2 cell monolayers [8–10]. Ganapathy and Leibach proposed a model to account for the interaction of H^+^-coupled di/tripeptide transport and Na^+^ absorption [11] whereby the intracellular acidification caused by di/tripeptide associated H^+^ influx activated an apical Na^+^/H^+^ exchanger and thus Na^+^ absorption. This model also explains the partial Na^+^-dependence of di/tripeptide uptake observed in mammalian small intestine and Caco-2 cell monolayers [11,12]. Inhibition of Na^+^/H^+^ exchange by removal of extracellular Na^+^, prevents the enterocyte from maintaining intracellular pH (pH_i_) during H^+^/solute influx and, therefore, abolishes the driving force (the transmembrane H^+^ electrochemical gradient) for further transport. The apical Na^+^/H^+^ exchanger specifically activated by di/tripeptide uptake is NHE3 (SLC9A3) which plays a significant role in Na^+^ absorption in the small intestine [7,10,13].

As hPepT1 is involved in both protein assimilation and drug delivery it is important to elucidate how altering hPepT1 function may affect nutritional status and oral bioavailability. Progress has been made in understanding how intestinal di/tripeptide transport is regulated by hormonal and neural signals as well as by disease states and surgical intervention (for review see [14]). However, little is known about how co-administered drugs or dietary factors affect hPepT1 function or the function of other brush-border membrane transport proteins. Alteration of cAMP levels within enterocytes by hormonal, neural and paracrine signals is a key step in the regulation of many brush-border membrane nutrient and electrolyte transporters. NHE3 is inhibited acutely by increasing [cAMP]_i_ [15]. Therefore, the aim of this study was to identify how dietary and orally-delivered compounds, which act as phosphodiesterase inhibitors (and thus increase [cAMP]_i_ by preventing breakdown), affect hPepT1 function. The dietary phosphodiesterase inhibitors caffeine and theophylline and the orally-delivered drug pentoxifylline all inhibit dipeptide uptake across the brush-border membrane of human intestinal epithelial Caco-2 cell monolayers. This inhibition is not a direct effect on hPepT1 but rather is indirect through inhibition of NHE3.

## 2 Materials and methods

### 2.1 Materials

Glycyl[1-^14^C]sarcosine (specific activity 57 mCi mmol^− 1^)] was obtained from Cambridge Research Biochemicals (Billingham, UK). [3-^3^H]β-Alanine (specific activity 50 Ci mmol^− 1^)] was obtained from American Radiolabeled Chemicals (St. Louis, MO, USA). PACAP (pituitary adenylate cyclase-activating polypeptide) was from Bachem (St. Helens, UK). Biotrak cAMP enzyme immunoassay was from GE Healthcare (Chalfont St. Giles, UK). 2′,7′-Bis(2-carboxyethyl-5(6)-carboxyfluorescein) acetoxymethyl ester (BCECF-AM) was from Invitrogen (Paisley, UK). Transwell polycarbonate filters were from Corning (Schiphol-Rijk, The Netherlands). S1611 was obtained from H.J. Lang (Aventis Pharma Deutschland GmbH, Frankfurt/Main, Germany). Forskolin, phosphodiesterase inhibitors, cell culture media and supplements were from Sigma-Aldrich (Poole, UK). All other chemicals were from Sigma-Aldrich or VWR (Lutterworth, UK) and were of the highest quality available.

### 2.2 Cell culture

Caco-2 cells (passages 100–119) were cultured as confluent monolayers on permeable polycarbonate filters, as described previously [16]. Monolayers were used at 14–22 days post-seeding and fed approximately 24 h prior to use.

### 2.3 Measurement of dipeptide uptake

[^14^C]Gly–Sar (0.5 μCi ml^− 1^, 0.1 – 10 mM) or [^3^H]β-alanine (0.5 μCi ml^− 1^, 0.1 mM) uptake across the apical membrane of Caco-2 cell monolayers was measured, essentially as described previously [16]. Briefly, Caco-2 cells were washed (in 4 × 500 ml) and bathed in modified Krebs' solution (composition (mM): NaCl, 137; KCl, 5.4; MgSO_4_, 0.99; KH_2_PO_4_, 0.34; NaH_2_PO_4_, 0.3; CaCl_2_, 2.8; Glucose, 10) or Na^+^-free solution (identical except choline chloride replaced NaCl and NaH_2_PO_4_ was omitted). Uptake was measured at apical pH 5.5 or pH 6.5 (10 mM MES, adjusted to the correct pH by Tris) with the basolateral solution being pH 7.4 (10 mM HEPES, adjusted to the correct pH by Tris) in all experiments. Uptake was measured over 15 min (37 °C) at the end of which the monolayers were rinsed thoroughly (in 3 × 500 ml, ice-cold, pH 7.4 Krebs' solution). Various compounds were added to the buffers for the duration of uptake (see figure legends for details). Cell monolayer-associated radioactivity was determined by scintillation counting.

### 2.4 Measurement of pH_i_

Intracellular pH (pH_i_) was measured in Caco-2 cell monolayers by microspectrofluorimetry using the pH-sensitive dye BCECF, essentially as described previously [10,17]. Cell monolayers were acidified by superfusion with apical Gly–Sar (20 mM, 5 min) in modified Krebs' solution (Na^+^-free, apical pH 5.5). The basolateral solution was Na^+^-free, pH 7.4 throughout all experiments. pH_i_ recovery was then measured in Na^+^-containing pH 7.4 apical solution until pH_i_ returned to baseline. The monolayers were incubated with caffeine (5 mM, apical and basolateral) for 10 min. The cells were then exposed to Gly–Sar once again but in the continued presence of caffeine (thus total exposure time to the compound before pH_i_ recovery was 15 min). pH_i_ recovery was then measured in the continued presence of caffeine. The initial rate of pH_i_ recovery is expressed as H^+^ efflux calculated from the gradient of the first 30 s of recovery [18].

### 2.5 Measurement of intracellular cAMP levels

Caco-2 cell monolayers were washed in 4 × 500 ml Krebs' solution (pH 7.4). Monolayers were incubated for 15 min in the presence or absence of caffeine, pentoxifylline, theophylline (all 5 mM), or forskolin (10 μM) at apical pH 6.5 and basolateral pH 7.4. Monolayers were then washed in 3 × 500 ml, ice-cold Krebs' (pH 7.4) and lysed in 500 μl lysis buffer (from the Biotrak enzyme immunoassay kit) for 10 min. [cAMP]_i_ was measured following the kit protocol.

### 2.6 Statistical analysis

Data are expressed as mean ± SEM unless stated otherwise. Statistical comparisons of mean values were made using paired two-tailed Student's *t*-test or one-way analysis of variance (ANOVA) (using the Tukey–Kramer or Bonferroni's multiple comparisons pos*t*-test) as appropriate. Significance was assumed if *p* < 0.05. Curves were fitted using FigP software.

## 3 Results

### 3.1 Inhibition of dipeptide uptake by caffeine is via inhibition of NHE3

**Fig. 1:**
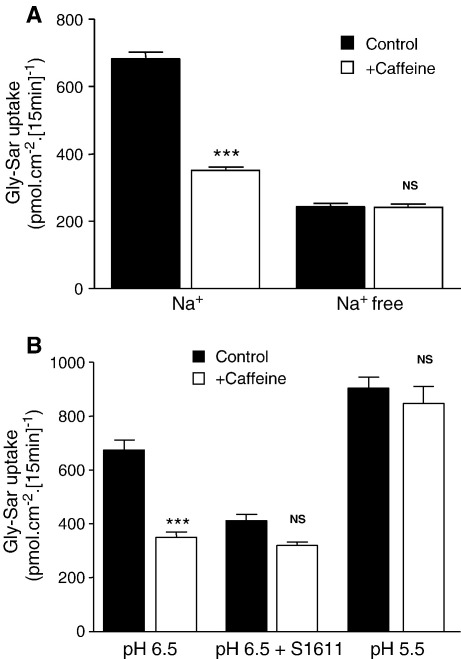
The effect of caffeine on dipeptide uptake across the apical membrane of Caco-2 cell monolayers. [^14^C]Gly–Sar (100 μM, 0.5 μCi ml^− 1^) uptake was measured (15 min, 37 °C) across the apical membrane of Caco-2 cell monolayers at apical pH 6.5 (unless stated otherwise) in the presence or absence of caffeine (5 mM, both apical and basolateral). Under all conditions basolateral pH was 7.4 (in the presence and absence of Na^+^, as appropriate). Results are expressed as mean ± SEM. (A) [^14^C]Gly–Sar uptake in the presence and absence of extracellular Na^+^ (*n* = 17–18). ***p < 0.001 vs. Na^+^ control; NS, *p* > 0.05 vs. Na^+^-free control. (B) [^14^C]Gly–Sar uptake at either apical pH 6.5 or pH 5.5 and in the presence and absence of the NHE3 inhibitor S1611 (3 μM, apical only) (*n* = 11–12). ***p < 0.001 vs. − S1611 control; NS, *p* > 0.05 vs. + S1611 control or vs. pH 5.5 control as appropriate.

Uptake of the dipeptide Gly–Sar (glycylsarcosine) across the apical membrane of Caco-2 cell monolayers was measured over 15 min at an apical pH (pH 6.5) representative of that found at the surface of the proximal small intestine. Incubation of Caco-2 cell monolayers with the dietary phosphodiesterase inhibitor caffeine (5 mM) at both the apical and basolateral surfaces (for the duration of the 15 min uptake period) reduced Gly–Sar uptake by around 50% (from 683.6 ± 18.9 to 351.1 ± 10.7 pmol cm^− 2^ [15 min]^− 1^, *n* = 17–18, *p* < 0.001, Fig. 1A). Caffeine was equally effective at inhibiting Gly–Sar uptake when added solely to either the apical or basolateral chamber (data not shown). Removal of extracellular Na^+^ reduces Gly–Sar uptake (as described previously [12]) (Fig. 1A). However, in the absence of extracellular Na^+^, caffeine had no effect on Gly–Sar uptake (uptake being 244.0 ± 9.1 and 242.0 ± 9.5 pmol cm^− 2^ [15 min]^− 1^ in the absence and presence of caffeine respectively, *n* = 17–18, *p* > 0.05).

Previously we have shown that the Na^+^-dependence of Gly–Sar uptake can be attributed to a lack of NHE3 activity in the absence of extracellular Na^+^ [10,12,18]. To test if the lack of effect of caffeine in the absence of extracellular Na^+^ was due to a requirement for NHE3 activity, dipeptide uptake was measured under other conditions where NHE3 would be inactive (Fig. 1B). At apical pH 6.5, the NHE3-selective inhibitor S1611 (3 μM) significantly reduced Gly–Sar uptake in a manner similar to that caused by the removal of extracellular Na^+^ (*p* < 0.001). This concentration of S1611 is sufficient to inhibit NHE3 completely but would not inhibit NHE2 or NHE1 [19]. Previous work has shown that in the absence of Na^+^, S1611 has no effect on Gly–Sar uptake into Caco-2 cells [12]. In addition, when hPepT1 is expressed in isolation in *Xenopus laevis* oocytes, dipeptide uptake is not inhibited by S1611 [7]. This observation confirms that the inhibition of dipeptide uptake by S1611 into Caco-2 cells is an indirect effect via inhibition of NHE3. In the presence of S1611, Gly–Sar uptake was no longer inhibited by caffeine (5 mM). The level of inhibition caused by caffeine alone, by S1611 alone and by both compounds together were not significantly different (*p* > 0.05) suggesting that caffeine and S1611 inhibit hPepT1 activity through a common mechanism (i.e. NHE3 inhibition). Gly–Sar uptake increases with decreasing apical pH, consistent with hPepT1 being a H^+^/dipeptide symporter. However, NHE3 activity decreases with acidification of the apical solution and is inactive by pH 5.5 due to the unfavourable transmembrane pH gradient [12]. Previous studies have shown that Gly–Sar uptake at apical pH 5.5 is not inhibited by S1611 or by the removal of extracellular Na^+^ [7,12]. Similarly, Fig. 1B shows that caffeine (5 mM) has no effect on Gly–Sar uptake at apical pH 5.5 (*p* > 0.05) suggesting again that caffeine is inhibiting Gly–Sar uptake through inhibition of NHE3.

### 3.2 The inhibition of hPepT1 by caffeine and related compounds is consistent with inhibition of phosphodiesterase activity

**Fig. 2:**
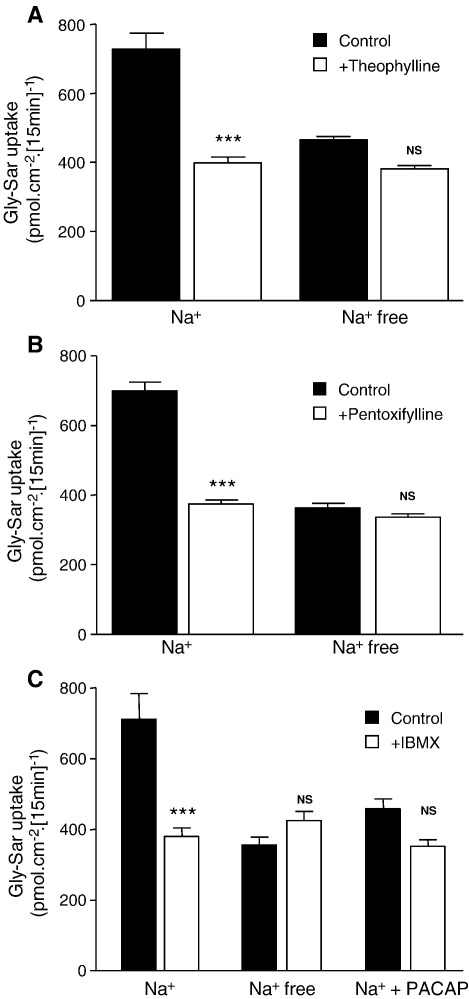
The effect of other phosphodiesterase inhibitors on dipeptide uptake. [^14^C]Gly–Sar (100 μM, 0.5 μCi ml^− 1^) uptake was measured (15 min, 37 °C) across the apical (pH 6.5) membrane of Caco-2 cell monolayers in the presence and absence of extracellular Na^+^. (A) [^14^C]Gly–Sar uptake in the presence and absence of theophylline (5 mM, both apical and basolateral) (*n* = 6). ***p < 0.001 vs. Na^+^ control; NS, *p* > 0.05 vs. Na^+^-free control. (B) [^14^C]Gly–Sar uptake in the presence and absence of pentoxifylline (5 mM, both apical and basolateral) (*n* = 12). ***p < 0.001 vs. Na^+^ control; NS, *p* > 0.05 vs. Na^+^-free control. (C) [^14^C]Gly–Sar uptake in presence and absence of IBMX (1 mM, both apical and basolateral, during the 15 min uptake and also for 1 h prior to uptake) and/or PACAP (5 nM, basolateral only, present during 15 min uptake only) (*n* = 12). ***p < 0.001 vs. Na^+^ control; NS, *p* > 0.05 vs. Na^+^-free control or vs. + PACAP control as appropriate.

Phosphodiesterase inhibitors structurally-related to caffeine and either found in diet (theophylline), or used as orally-delivered therapeutics (pentoxifylline) or used as laboratory tools (IBMX, 3-isobutyl-1-methylxanthine) were tested for inhibition of hPepT1 (Fig. 2). Theophylline (5 mM, Fig. 2A), pentoxifylline (5 mM, Fig. 2B) and IBMX (1 mM, Fig. 2C) reduced dipeptide uptake in the presence of extracellular Na^+^ (*p* < 0.001) but not in Na^+^-free conditions (*p* > 0.05). Previously, we have shown that the enteric neuropeptide PACAP (pituitary adenylate cyclase-activating polypeptide) and the related neuropeptide VIP (vasoactive intestinal peptide) reduce dipeptide uptake across the apical membrane of Caco-2 cell monolayers by inhibition of NHE3 [18]. PACAP activates the VPAC_1_ receptor which is coupled to adenylate cyclase and, therefore, increases [cAMP]_i_. Fig. 2C shows that when Caco-2 cell monolayers were incubated with PACAP (5 nM) in the basolateral solution for the duration of uptake, Gly–Sar uptake was reduced. Incubation of the cells with both PACAP and IBMX resulted in a reduction in uptake which was not significantly different from the uptake in the presence of either PACAP or IBMX alone suggesting IBMX, like PACAP, is inhibiting the NHE3-dependent component of Gly–Sar uptake.

Phosphodiesterase inhibitors increase the levels of cAMP within cells by preventing cAMP breakdown. However, caffeine can also elicit cell signalling effects such as antagonism of adenosine receptors and activation of calcium release from intracellular stores [20–22]. To confirm that the compounds used in this study inhibit hPepT1 function through increasing cAMP levels, [cAMP]_i_ in Caco-2 cell monolayers was measured. Monolayers were incubated with caffeine (5 mM), theophylline (5 mM), pentoxifylline (5 mM) or the adenylate cyclase activator forskolin (10 μM) which can also inhibit dipeptide uptake [12]. Cells were lysed and [cAMP]_i_ measured by enzyme immunoassay. Caffeine, theophylline and pentoxifylline caused [cAMP]_i_ to more than double from control levels of 950 ± 230 fmol cm^− 2^ (mean ± SD, *n* = 3) to 2200 ± 830, 2780 ±300 and 2330 ± 150 fmol cm^− 2^ (mean ± SD, *n* = 3), respectively. However, the change in cAMP was modest compared to [cAMP]_i_ detected in the presence of forskolin (92500 ±12990 fmol cm^− 2^ [mean ± SD, *n* = 3]).

**Fig. 3:**
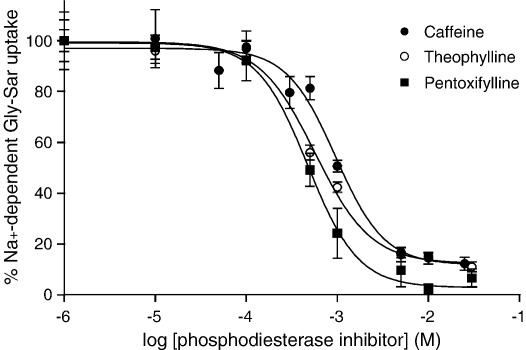
Concentration dependent inhibition of dipeptide uptake by caffeine, theophylline and pentoxifylline. [^14^C]Gly–Sar (100 μM, 0.5 μCi ml^− 1^) uptake was measured (15 min, 37 °C) across the apical (pH 6.5) membrane of Caco-2 cell monolayers in the absence and presence of caffeine (filled circles, 0.001–25 mM), theophylline (open circles, 0.001–30 mM) or pentoxifylline (filled squares, 0.001 – 30 mM). The results are presented as the percentage uptake in Na^+^-containing conditions (after subtraction of uptake in Na^+^-free conditions). Best-fit concentration response curves were fitted using FigP (*r*^2^ = 0.986, 0.994 and 0.996, for caffeine, theophylline and pentoxifylline, respectively). Data are mean ± SEM (*n* = 6–12).

Inhibition of the Na^+^-dependent (NHE3-dependent) component of Gly–Sar uptake by phosphodiesterase inhibitors was concentration-dependent (Fig. 3). The IC_50_ values were 1.0 ±0.2 mM for caffeine, 0.6 ± 0.1 mM for theophylline and 0.5 ±0.1 mM for pentoxifylline. The sensitivity of hPepT1 to these compounds is consistent with the effect being through an inhibition of phosphodiesterase activity [20,21,23–25]. At a concentration of 5 mM all three phosphodiesterase inhibitors almost completely inhibited Na^+^-dependent Gly–Sar uptake.

### 3.3 Pentoxifylline decreases the maximal capacity for dipeptide uptake

**Fig. 4:**
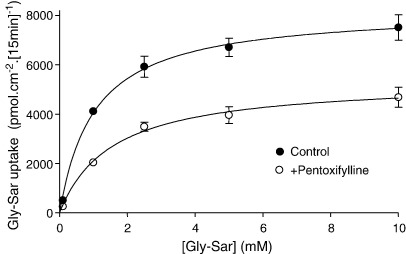
The effect of pentoxifylline on the concentration-dependent uptake of the dipeptide Gly–Sar across the apical membrane of Caco-2 cell monolayers. [^14^C]Gly–Sar (0.1–10 mM, 0.5 μCi ml^− 1^) uptake was measured (15 min, 37 °C) across the apical (pH 6.5) membrane of Caco-2 cell monolayers in the absence (control, filled circles) and presence (open circles) of pentoxifylline (5 mM). Lines are the best-fit curves for the hyperbolic Michaelis–Menten kinetics (carrier-mediated (saturable) uptake following subtraction of the linear component) fitted using FigP (*r*^2^ = 0.998 and 0.995 for control and + pentoxifylline, respectively). Results are mean ± SEM (*n* = 6).

Gly–Sar uptake across the apical membrane was measured at apical pH 6.5 at various concentrations of Gly–Sar (Fig. 4). In the presence of pentoxifylline (5 mM) Gly–Sar uptake was reduced compared to control at all Gly–Sar concentrations. Analysis of the Michaelis–Menten kinetics confirmed that the maximal capacity (*V*_max_) for Gly–Sar uptake was significantly reduced from 8246 ± 913 to 5369 ± 117 pmol cm^− 2^ [15 min]^− 1^ (*p* < 0.05, *n* = 6) by pentoxifylline but the affinity (*K*_m_) was not affected (1.0 ± 0.2 mM and 1.6 ± 0.6 mM in the absence and presence of pentoxifylline, respectively, *p* > 0.05). Reduction in the capacity for dipeptide uptake is consistent with a reduction in the driving force (the H^+^-electrochemical gradient) for uptake through inhibition of NHE3. Previously, we have shown that S1611 and forskolin similarly decrease the *V*_max_ for dipeptide uptake across the apical membrane of Caco-2 cell monolayers [12].

### 3.4 Caffeine inhibits pH_i_ recovery (NHE3 function) after dipeptide-induced acidification

**Fig. 5:**
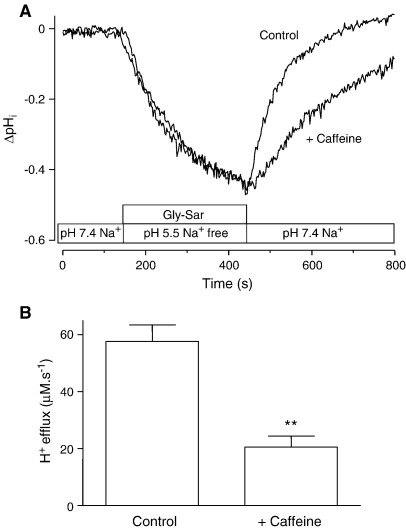
Caffeine inhibits NHE3-mediated pH_i_ recovery. Caco-2 cell monolayers loaded with the pH-sensitive dye BCECF were allowed to equilibrate in Na^+^-containing pH 7.4 solution before being exposed to Gly–Sar (20 mM) at the apical membrane for 5 min in Na^+^-free, pH 5.5 solution (basolateral solution was Na^+^-free, pH 7.4 throughout experiment). After removal of Gly–Sar, pH_i_ recovered rapidly back to baseline levels in Na^+^-containing pH 7.4 apical solution. The monolayers were then incubated with caffeine (5 mM, both apical and basolateral) for 10 min before Gly–Sar induced acidification and the subsequent pH_i_ recovery were again measured (in the continued presence of caffeine). A. Representative trace showing the composite responses of a single monolayer in the absence (Control) and presence (+ Caffeine) of caffeine. The horizontal bars identify the composition of the apical bathing solution. B. H^+^ efflux was calculated from the mean rate of change of pH_i_ over the initial 30 s of pH_i_ recovery from Gly–Sar induced acidification in the presence (+ Caffeine) and absence (Control) of caffeine. Data are mean ± SEM (*n* = 4) and statistical analysis was performed using Student's *t*-test: **p < 0.01 vs. Control.

Previous work demonstrated that NHE3 (but not NHE1 or NHE2) is selectively activated by the intracellular acidification associated with H^+^/dipeptide symport in Caco-2 cell monolayers [10,12]. S1611, the removal of extracellular Na^+^, or factors that increase [cAMP]_i_, such as forskolin and VIP, all inhibit the ability of the cell to regulate pH_i_ after H^+^/solute-induced intracellular acidification [10,12,18,26]. pH_i_ was measured using the pH-sensitive dye BCECF (Fig. 5). Caco-2 cell monolayers loaded with BCECF were superfused across the apical membrane with Na^+^-containing, pH 7.4 modified Krebs' solution. The basolateral solution was maintained as Na^+^-free, pH 7.4 solution throughout the experiment. Cells were exposed to Gly–Sar (20 mM) under conditions where hPepT1 activity would be maximal but where there would be no concurrent NHE3 activity (pH 5.5, Na^+^-free). After 5 min, Gly–Sar had induced a large intracellular acidification. When Gly–Sar was removed and the apical solution returned to a Na^+^-containing, pH 7.4 solution, pH_i_ recovered rapidly back to baseline levels. The same monolayers were then incubated with caffeine (5 mM) in both the apical and basolateral solutions for 10 min. Gly–Sar was then reintroduced for 5 min in the continued presence of caffeine. The degree and rate of acidification caused by Gly–Sar was not altered by caffeine suggesting that hPepT1 is not directly regulated by phosphodiesterase inhibitors. However, the ability of the cell to recover pH_i_ after Gly–Sar-induced acidification was attenuated after the 15 min exposure to caffeine (Fig. 5A). The degree of attenuation was quantified by calculating the H^+^ efflux rate over the first 30 s of pH_i_ recovery (Fig. 5B). In the presence of caffeine H^+^ efflux was reduced from 57.6 ± 5.8 to 20.5 ± 3.9 μM s^− 1^ (*n* = 4, *p* < 0.01).

To confirm that phosphodiesterase inhibitors do not have any direct inhibitory effect on dipeptide transport, hPepT1 was expressed in *Xenopus laevis* oocytes, as described previously [7]. Incubation of hPepT1-expressing oocytes with phosphodiesterase inhibitors such as caffeine (5 mM) had no effect on dipeptide uptake (data not shown).

### 3.5 Inhibition of NHE3 by caffeine also inhibits the H^+^/amino acid transporter hPAT1 (SLC36A1)

**Fig. 6:**
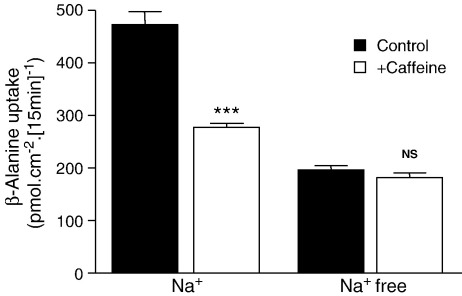
The effect of caffeine on amino acid uptake via hPAT1 across the apical membrane of Caco-2 cell monolayers. [^3^H]β-Alanine (100 μM, 0.5 μCi ml^− 1^) uptake was measured (15 min, 37 °C) across the apical membrane of Caco-2 cell monolayers at apical pH 6.5 in the presence or absence of Na^+^ and the presence or absence of caffeine (5 mM, both apical and basolateral). Basolateral pH was 7.4 (in the presence and absence of Na^+^ and caffeine, as appropriate). Results are expressed as mean ± SEM (*n* = 12). ***p < 0.001 vs. Na^+^ control; NS, *p* > 0.05 vs. Na^+^-free control.

The indirect nature of the inhibition of hPepT1 by phosphodiesterase inhibitors suggests that any apical solute transporter dependent upon the transmembrane H^+^-electrochemical gradient will similarly be regulated by these compounds. The H^+^-coupled amino acid transporter hPAT1 (SLC36A1) has been isolated from Caco-2 cell monolayers [27]. As well as mediating the uptake of a wide variety of amino acids, hPAT1 can also transport orally-active drugs such as the anti-epileptic vigabatrin [28]. Previously we have identified that amino acid uptake into hPAT1-expressing oocytes is Na^+^-independent but hPAT1-mediated amino acid uptake into Caco-2 cells is partially Na^+^-dependent [26,29,30]. Intracellular acidification caused by the hPAT1 substrate β-alanine selectively activated Na^+^/H^+^ exchange by NHE3 [26]. Like H^+^-coupled dipeptide uptake, H^+^-coupled amino acid uptake into Caco-2 cells is inhibited by forskolin, S1611 and VIP in a Na^+^ and pH-dependent manner via inhibition of NHE3 [26,29,30]. Uptake of the hPAT1 substrate β-alanine [16] was measured across the apical membrane of Caco-2 cell monolayers at apical pH 6.5 for 15 min (Fig. 6). Caffeine (5 mM) reduced β-alanine uptake in the presence (*p* < 0.001) but not the absence of extracellular Na^+^ (*p* > 0.05) suggesting that H^+^-coupled amino acid uptake via hPAT1 is also modulated indirectly through regulation of NHE3.

## 4 Discussion

The di/tripeptide transporter hPepT1 acts as a high-capacity route for solutes across the first barrier to oral-bioavailability, the brush-border membrane of the small intestine. Many, orally-active peptidomimetics and amino acid-conjugated pro-drugs have been identified as hPepT1 substrates [3,4]. There is an increasing number of examples of physiological regulation (hormonal, neural, paracrine) of hPepT1 and of regulation of hPepT1 in certain disease states and after surgery (reviewed by [14]). Another, less studied, factor which may affect the degree to which drugs are absorbed across the small intestinal epithelium is interaction with co-administered drugs or components of diet. Exposure of Caco-2 cell monolayers to the hPepT1 substrate Gly–Gln for 4 days resulted in a subsequent increase in capacity for dipeptide uptake and in hPepT1 expression [31]. Another study found that an array of flavonoids, which are found ubiquitously in foods of plant origin, either inhibit, have no effect or increase the hPepT1-mediated uptake of the antibiotic cefixime into Caco-2 cell monolayers [32]. In this study we identify that incubation of human intestinal epithelial cells with either dietary or orally-active therapeutic phosphodiesterase inhibitors reduces Gly–Sar uptake through a reduction in hPepT1 capacity.

The data presented here show that the inhibition of Gly–Sar uptake by phosphodiesterase inhibitors is both Na^+^- and pH-dependent (Figs. 1 and 2) suggesting that inhibition is not a direct effect on hPepT1 but rather through NHE3. When NHE3 is inhibited (e.g. by the removal of extracellular Na^+^ or by addition of S1611) the cells are no longer able to maintain pH_i_ during solute-induced acidification and, therefore, the driving force (the transmembrane H^+^ electrochemical gradient) for further dipeptide uptake is reduced. Previously, we have shown that hPepT1 can be inhibited by other factors which are known to increase cAMP in intestinal epithelial cells such as the enteric neuropeptides VIP and PACAP [18]. Although caffeine, theophylline and pentoxifylline can elicit effects through pathways other than increasing cAMP, a number of factors suggest that they are acting here as phosphodiesterase inhibitors. Firstly, incubating Caco-2 cell monolayers with all three compounds produced an increase in [cAMP]_i_. The increase is relatively small compared to that produced by forskolin. However, this could be due to the cAMP signal being compartmentalised (as demonstrated in cardiomyocytes [33]) and thus the local change may be much greater than the measured, global change. Secondly, phosphodiesterases can also regulate the levels of cGMP but none of the compounds tested here produced a significant change in [cGMP]_i_ (data not shown). Lastly, the concentrations of caffeine, theophylline and pentoxifylline required to produce inhibition of hPepT1 (IC_50_ = 1.0, 0.6 and 0.5 mM, respectively, Fig. 3) are much closer to those for inhibition of phosphodiesterases than for other effects such as antagonism of adenosine receptors (IC_50_≈ 2–80 μM) and release of intracellular calcium (EC_50_≈ 5–20 mM) [20–22,34–36]. Theophylline (100 μM) has been shown to abolish increased glucose absorption resulting from activation of A_2_ receptors by luminal adenosine (5 mM) in mouse small intestine in vivo [37]. However, in addition to the significantly higher affinity for theophylline, A_2_ receptors are positively coupled to adenylate cyclase so antagonism of the receptor should cause a decrease in [cAMP]_i_ and not the increase noted here [22,37].

Caffeine (and to a lesser extent theophylline) is found in high levels in many beverages either naturally or by addition. For example, the average cup of coffee contains 85 mg of caffeine [38]. Pentoxifylline is given orally as 400 mg tablets to treat intermittent claudication as part of peripheral arterial disease [39]. Although it is difficult to predict the actual concentration at the surface of the small intestinal epithelium it is conceivable that caffeine and pentoxifylline may reach concentrations high enough to elicit an effect on hPepT1 similar to that measured here using Caco-2 cell monolayers. There is evidence that caffeine given orally in doses equivalent to diet can elicit changes in small intestinal function. A study using human volunteers found that ingestion or intraluminal jejunal perfusion of caffeine (75–300 mg) caused a rapid reversal of net fluid absorption to net secretion [40]. Although the cell signalling pathway by which caffeine stimulated fluid secretion was not investigated, this study does indicate that the concentrations of caffeine used here in vitro may be sufficient to cause changes in the small intestinal epithelium in vivo.

Since the initial reports of phosphodiesterase activity it has become evident that there are many types of phosphodiesterase. Currently there are 11 different PDE families each of which can contain several isoforms and splice variants [24,41]. Many of these isoforms are tissue or cell-specific and recent studies have shown that they can be expressed in distinct sub-cellular locations where they act to limit site, amplitude and duration of cyclic nucleotide signals [24,33]. Little is known about which PDE isoforms are found in the small intestinal epithelium. Studies reporting tissue distribution of PDE mRNA expression often include a small intestinal sample but generally it is not clear whether the PDE is expressed specifically in enterocytes. A single study of PDE3 isoforms identified that PDE3B but not PDE3A mRNA was highly abundant in the epithelium and underlying smooth muscle layers of developing small intestine of rat [42].

Caffeine, theophylline, IBMX and pentoxifylline generally inhibit all PDE isoforms with roughly equal efficacy [25,43,44]. However, since many PDE isoforms and variants are located in distinctive cell and sub-cellular locations they are now considered to be good targets for specific therapeutic agents. Often the resulting drugs are given orally, for example, the PDE5 inhibitor sildenafil used to treat erectile dysfunction [45]. PDE5 is expressed at the mRNA level in the small intestine [46,47]. Further work is required to identify which PDE isoforms are expressed in small intestinal epithelial cells both along the proximal–distal and crypt–villus axes and which isoforms play a functional role in regulating solute transport at the brush-border membrane.

In conclusion, we have shown that non-nutrient components of diet and orally-delivered therapeutic agents both regulate activity of the nutrient and drug transporter hPepT1. This regulation is an indirect effect through inhibition of apical NHE3 function, which acts as a pH homeostatic mechanism during H^+^-coupled dipeptide transport. Functional cooperativity between hPepT1 and NHE3 results not only in the absorption of di/tripeptides but also stimulation of Na^+^ absorption [8–10]. The H^+^-coupled amino acid transporter hPAT1 (SLC36A1) is regulated by caffeine in a similar, indirect manner to hPepT1 (Fig. 6). Thus, through modulation of a single homeostatic mechanism (NHE3), a luminal compound may influence several different absorptive mechanisms. The results presented here suggest that any regulation of NHE3 activity by luminal contents, co-administered drugs or by neurohormonal signals may have significant impact on the general absorptive state of the cell. Any other nutrient transporter dependent on the maintenance of the H^+^ electrochemical gradient for function may be regulated in a similar manner.
